# Epigenetic modifications in breast cancer: from immune escape mechanisms to therapeutic target discovery

**DOI:** 10.3389/fimmu.2025.1584087

**Published:** 2025-04-17

**Authors:** Ziyu Kang, Junlin Wang, Jiyan Liu, Li Du, Xiaofei Liu

**Affiliations:** ^1^ Institute of Chinese Medical Literature and Culture, Shandong University of Chinese Medicine, Jinan, China; ^2^ Department of Pharmacy, Shandong Second Provincial General Hospital, Jinan, China; ^3^ Pharmacy Department, Jinan Licheng District Mental Health Prevention and Control Center, Jinan, China; ^4^ Department of Acupuncture and Moxibustion, Shandong College of Traditional Chinese Medicine, Yantai, China; ^5^ Breast and Thyroid Surgery, Shandong University of Traditional Chinese Medicine Affiliated Hospital, Jinan, China

**Keywords:** breast cancer, epigenetic modifications, immune escape, multi-omics, therapeutic targets

## Abstract

Breast cancer (BC) is one of the most prevalent malignant tumors among women globally, with the number of cases accounting for even more than 1/3 of all tumor patients in women. Recent studies have found that the incidence of BC is increasing every year. Despite the great progress made in BC treatment, the characteristics of BC cells, such as strong immune evasion, easy recurrence and drug resistance, are still the main reasons limiting the survival of BC patients. Epigenetics is becoming an important method to reveal the development of cancer, mainly through the study of DNA methylation, histone modification, chromatin structure changes and non-coding RNA. In addition, researchers have found that epigenetic markers have great potential for early detection and personalized treatment of BC. Inhibitors targeting epigenetically modified enzymes are effective in treating a wide range of tumors and provide significant patient survival and quality of life. Therefore, this review will comprehensively summarize the role of epigenetic modifications in BC development. Second, this paper will focus on summarizing how epigenetic modifications induce the formation of tumor immune microenvironment (TIME) in BC. Targeting the mechanism of action of epigenetic modifications provides new perspectives to unravel the complex process of BC development, while paving the way for the development of novel diagnostic and therapeutic targets. In the future, by integrating multi-omics data to enable a deeper understanding of the pathogenesis of BC, we will be able to promote the overall development of precision medicine.

## 1 Introduction

BC is one of the most common malignant tumors affecting the lives and health of women worldwide (1). Global cancer statistics show that BC claims hundreds of thousands of women’s lives every year, and its incidence and mortality rates are still on the rise, especially in high-income regions (2). As of 2022, there are already more than 2.3 million new cases of BC worldwide, and by 2025 that number will double. Among women with tumors, the incidence of breast cancer is 1/3. Unfortunately, about 15% of BC patients die each year due to ineffective treatment. BC places a huge burden on the world (3, 4). Currently, with the gradual spread of early detection and continuous advances in treatment methods, among others, the overall survival rate of patients has gradually improved (5–7). However, many patients still face major challenges in terms of prognosis: including drug resistance, growth recurrence and metastasis (8–10). The ability of BC cells to effectively evade recognition and killing by the immune system is another major factor contributing to the poor quality of patient survival. These clinical issues suggest that breast cancer growth is not only driven by genetic mutations, but may also involve more complex molecular mechanisms. Epigenetics is the study of how genes are turned on or off without altering the DNA itself. In recent years, changes in epigenetic modifications have played an important role in BC (11, 12). These regulatory mechanisms influence important processes such as tumor cell proliferation, apoptosis and immune escape by regulating gene expression. In BC, epigenetic modifications are regulated in multiple ways (12–14). These epigenetic changes are closely associated with the onset and progression of BC. They may also be a key cause of tumor immune escape, ineffective treatment and cancer recurrence (15).

Epigenetic research has actually been relocating in the direction of professional applications, showing wonderful possible, especially in the fields of early lump diagnosis and customized treatment. The growth of liquid biopsy modern technology has made it possible for the non-invasive detection of epigenetic pens, such as methylated DNA or non-coding RNA in blood or other body fluids, permitting very early BC diagnosis (16, 17). Additionally, inhibitors targeting epigenetic modification enzymes have actually demonstrated therapeutic possibility in a number of kinds of cancer cells (18). Despite significant progress in epigenetic studies of BC, many challenges remain to be addressed. How precisely epigenetic modifications regulate the growth, proliferation, and function of tumor cells and immune cells, and how to develop novel therapeutic targets against epigenetic modifications are major challenges for future research.

Epigenetics provides a fresh perspective to understand the complexity of BC and creates new opportunities for the development of its novel therapeutic targets. This article provides insight into the mechanisms by which epigenetic abnormalities play a role in BC. Meanwhile, its role in promoting BC immune escape is summarized in focus. Finally, we summarize the current progress in the development of epigenetic modifier enzyme inhibitors and their potential in immunotherapy combination applications. In the future, we will lay a solid theoretical foundation for the development of novel therapeutic targets and treatment regimens for BC.

## 2 Epigenetics definition and basic types

Epigenetics focuses on how genes are turned on or off without altering their own DNA sequence. These regulatory mechanisms include chemical modifications and other molecular processes (19). Major epigenetic changes include DNA methylation, the way chromatin is organized, and the role of noncoding RNAs.

DNA methylation frequently takes place at CpG islands, where the addition of methyl teams prevents gene expression (20). DNA methylation, the most classical form of epigenetic modification, is catalyzed by DNA methyltransferases, which add methyl groups to cytosines at CpG islands and usually inhibit gene transcription. In tumor cells, hypermethylation of oncogene promoters can lead to their silencing, thereby promoting tumor proliferation and metastasis. Changes to histones, like adding or removing chemical groups on their tails, affect how tightly DNA wraps around them. This controls how accessible and active the genes are (21). For example, histone acetylation loosens chromatin to promote transcription by neutralizing charge, while methylation (e.g., H3K27me3) can repress oncogene expression. Chromatin remodeling alters the framework of chromatin, ensuring genes more or less obtainable for transcription (22). Non-coding RNAs (NcRNAs), like miRNAs and lncRNAs, can control how genes work by attaching to mRNAs or by affecting transcription factors (23). Non-coding RNAs (e.g. miRNAs, lncRNAs) regulate gene expression by targeting mRNA degradation, recruiting epimerase modifying enzymes, or competitive binding, e.g. miR-21 targets oncogenes, whereas HOTAIR lncRNA promotes oncogene activation by recruiting EZH2 (24). Recent developments in epigenetics study have actually highlighted the crucial duties of these adjustments in various pathological problems, including cancer, metabolic diseases, neurological disorders, and immune diseases (25). Particularly, epigenetic adjustments are considered crucial regulatory factors in tumorigenesis and development. Recent studies have actually demonstrated that these alterations are reversible (26, 27), giving a crucial structure for developing unique healing techniques. As a result, the development of epigenetic drugs has become a significant emphasis in cancer treatment. Epigenetic modifications play a crucial role in the development of BC (28).

## 3 Mechanisms of epigenetic abnormalities in BC

### 3.1 DNA methylation

DNA methylation is an epigenetic device largely happening at CpG islands, which are areas rich in cytosine and guanine dinucleotides. This process involves adding a methyl group to the fifth carbon of the cytosine ring using enzymes called DNA methyltransferases (DNMTs). When CpG islands located in gene promoter regions are methylated, it commonly leads to the transcriptional silencing of the affiliated genetics, consequently properly hindering their expression. In growth cells, the DNA methylation standing is often abnormal, defined by a global decrease in methylation degrees that results in enhanced genetic instability. *De novo* methylation primarily occurs in the promoter regions of tissue-specific genes. Oncogenes are frequently hypomethylated, leading to their aberrant activation, whereas tumor suppressor genes are often hypermethylated, resulting in transcriptional silencing. These problems collectively affect various elements of BC cell behavior, including expansion, apoptosis, invasion, metastasis, and feedback to treatment (29).

Genes that stop tumors, like breast cancer susceptibility gene 1(BRCA1), p16, and RASSF1A, often have high levels of methylation in the CpG islands near their starting points. This makes these genes inactive. The inactivation of these genetics promotes cell spreading, hinders apoptosis, and promotes tumor formation and advancement (30). Studies have actually shown that in BC individuals, hypermethylation of the BRCA1 marketer directly causes a significant decrease in gene expression, a sensation observed in as much as 30% of erratic BC situations (31). A study team at Lund University in Sweden (32) used multi-omics data from BC to assess the frequency of BRCA1 promoter hypermethylation and functional loss characteristics in early triple-negative BC (TNBC). They discovered that the incidence of BRCA1 hypermethylation in early unclassified TNBC is two times that of useful loss lumps, suggesting a solid connection with very early TNBC. Furthermore, hypermethylation of the p16 gene is associated with the unlimited proliferation of BC cells, and increased methylation levels are closely related to dysregulation of cell cycle control (33). Abnormal DNA methylation may also indirectly lead to the overexpression of oncogenes through complex epigenetic regulatory networks. For instance, in BC, the ERα gene often has less methylation, which leads to it being more active. This higher activity is linked to the cancer getting worse and becoming more invasive (26). More and more studies show that DNA methylation plays a role in how cancer starts and could be used as a sign for early detection of BC. But, using just one gene’s methylation isn’t very accurate for diagnosis, so it’s better to look at several places together for a better prediction.

### 3.2 Abnormal histone modifications

Histones are very basic proteins that have a lot of lysine and arginine. They are found in the nuclei of eukaryotic cells. These act like spools for DNA to wind around, making nucleosomes. This helps stop DNA from getting tangled or damaged. It also has an important part in controlling genes and copying DNA.

Histone modifications, particularly methylation, acetylation, and phosphorylation, are critical in tumorigenesis. Histone methylation changes how genes are expressed by changing the chromatin structure. The place and amount of methylation can lead to different effects on genes. For example, when there is a lot of methylation on lysine 4 of histone H3 (H3K4me3), it usually means the gene activation. This adjustment keeps chromatin in a reasonably open state, permitting transcription factors and RNA polymerase to accessibility DNA, consequently promoting gene expression (34). On the other hand, when there is a lot of methylation on lysine 9 and lysine 27 of histone H3, it usually means gene silencing. These modifications attract silencing complicated, leading to tighter chromatin packaging and preventing gene expression (35). For instance, in BC cells, the H3K27me3 level at the promoter areas of tumor suppressor genes like BRCA1 is substantially elevated. This methylation recruits the PRC2, additional tightening chromatin framework and stopping the binding of transcription factors, consequently inhibiting the expression of tumor suppressor genes (36). High acetylation of lysine residues at the amino termini of histones is typically connected with open chromatin and gene transcription activation, while low acetylation is associated with genetics silencing or repression (37).Histone acetylation levels are crucial in the development of various tumor types, including thyroid cancer (38), BC (39), cervical cancer (40), and prostate cancer (41). In BC, HAT1 from the GNAT family induces histone H3 acetylation at the CCR4 promoter in Tregs through the FOXP3/HAT1 complex, promoting Tregs infiltration in the tumor microenvironment and tumor cell immune evasion (42). MOF, a member of the MYST family of acetyltransferases specific for lysine 16 on histone H4, is upregulated in BC, leading to overexpression of H4K16ac, resulting in dysregulated DNA damage response and cell growth in tumor cells (43, 44). The balance of histone acetylation is regulated by HATs and HDACs. An imbalance can lead to modification disorders and tumorigenesis. HDAC inhibitors are potential therapeutic agents for BC. Studies have found that HDAC1 (45), HDAC2 (46), and HDAC3 (47, 48) are significantly overexpressed in BC, with high expression levels positively correlated with advanced TNM and N stages and negatively correlated with DFS and OS, serving as independent prognostic factors. However, in Asian BC patients, increased HDAC1 expression seems to longer OS (37). Overexpression of SIRT6 can inhibit Tbx3 expression through deacetylation of lysine 9 on histone H3. Loss-of-function mutations or low expression of Tbx3 can predict poor prognosis in HER2-positive BC patients (49). The abnormal expression and function of HDACs in BC suggest their potential as novel indicators of invasiveness and therapeutic targets.

### 3.3 Abnormal expression of non-coding RNA

NcRNAs are very important in starting and developing BC. When ncRNAs are not expressed normally, they can affect how BC develops in different ways. Both types of RNAs help regulate the function of genes.

miRNAs control genes by attaching to the 3’ UTR of target mRNAs. This stops the mRNAs from being translated into proteins or causes them to break down. In BC, miR-21 is a well-known example. Its overexpression targets and inhibits the tumor suppressor gene PTEN, leading to the abnormal activation of the PI3K/AKT signaling pathway, which promotes cell proliferation and survival (35). Research has shown that many miRNAs specifically regulate the expression of target genes and signaling pathways in BC stem cells (BCSCs) (50). These miRNAs are essential for the self-renewal, growth, and metastasis of BC cells and can serve as potential diagnostic markers for tumor progression, metastasis, and therapeutic response. For instance, the overexpression of miR-155 is associated with the aggressiveness and malignancy of BC. It targets and inhibits the cytokine signaling inhibitor SOCS1, activates the JAK/STAT signaling pathway, and enhances the proliferation and metastatic potential of cancer cells (36).

LncRNAs are a type of RNA that is more than 200 nucleotides long and does not make proteins. They were once considered “transcriptional noise” without biological function. However, with the advancement of high-throughput technologies and in-depth research, lncRNAs have been found to have significant biological functions under physiological conditions. lncRNAs influence BC development by encoding peptides (51, 52), regulating epigenetics (53, 54), modulating immunity (55), and regulating protein expression (56). Key lncRNAs associated with BC include H19, which promotes cancer cell proliferation, and MALAT1, which facilitates distant metastasis of cancer cells. High levels of H19 expression in BC cells are associated with positive expression of the tumor HER2 (57). H19 promotes cancer cell migration and can serve as a potential biomarker for BC diagnosis (58). Studies have found that MALAT1 is associated with cancer invasion and metastasis (59). Therefore, MALAT1 can promote the distant metastasis of BC cells (60). Researchers have discovered that MALAT1 can inhibit the distant metastasis of cancer cells in triple-negative BC patients by regulating HIF-1α (61). Additionally, clinical studies have shown that certain lncRNAs, which inhibit the migration of cancer cells in tumor suppressor genes, such as MAGI2-AS3, are significantly downregulated in BC tissues. The expression level of MAGI2-AS3 is linked to the tumor spreading to lymph nodes (62). In patients with triple-negative BC, there is much less MAGI2-AS3, and this is related to how long patients stay free of the disease (63). MAGI2-AS3 is expected to be an effective indicator for assessing patient prognosis and recurrence, and for use in individualized tumor therapy (**Table 1**).

**Table 1:** Epigenetic markers in BC subtypes.

Breast Cancer Subtype	Epigenetic Marker	Gene Expression	Clinical Relevance	Therapeutic Significance
Luminal A	Hypermethylation of ESR1	Decreased ESR1 expression	Favorable prognosis	Responsive to endocrine therapy
Luminal A	Hypermethylation of GATA3	Decreased GATA3 expression	Favorable prognosis	Responsive to endocrine therapy
Luminal B	Hypermethylation of CCND1	Increased CCND1 expression	Less favorable prognosis compared to Luminal A	Combination of endocrine therapy and chemotherapy
Luminal B	Hypermethylation of PR	Decreased PR expression	Prognosis better than Luminal A	Potential resistance to endocrine therapy
HER2-positive Breast Cancer	Overexpression of miR-21	Increased HER2 expression	Rapid progression and poor prognosis	Effective in HER2-targeted therapy
HER2-positive Breast Cancer	Hypermethylation of RASSF1A	Reduced RASSF1A expression	Higher risk of metastasis	Potential application of miRNA-based therapies
Triple-negative Breast Cancer	Hypermethylation of BRCA1	Reduced BRCA1 expression	Elevated risk of disease progression	Responsive to platinum-based chemotherapy
Triple-negative Breast Cancer	Loss of H3K27me3	Decreased genome-wide H3K27me3 levels	Increased sensitivity to chemotherapy	Potential benefit from HDAC inhibitors
Basal-like Breast Cancer	Hypermethylation of TP53	Decreased TP53 expression	Highly aggressive with poor prognosis	Responsive to platinum-based chemotherapy
Basal-like Breast Cancer	Hypermethylation of CDH1	Decreased E-cadherin	Loss of cell adhesion leading to increased invasiveness	Potential for epigenetic therapy

## 4 Epigenetic modifications remodel the immune-suppressive microenvironment in BC

**Figure 1:**
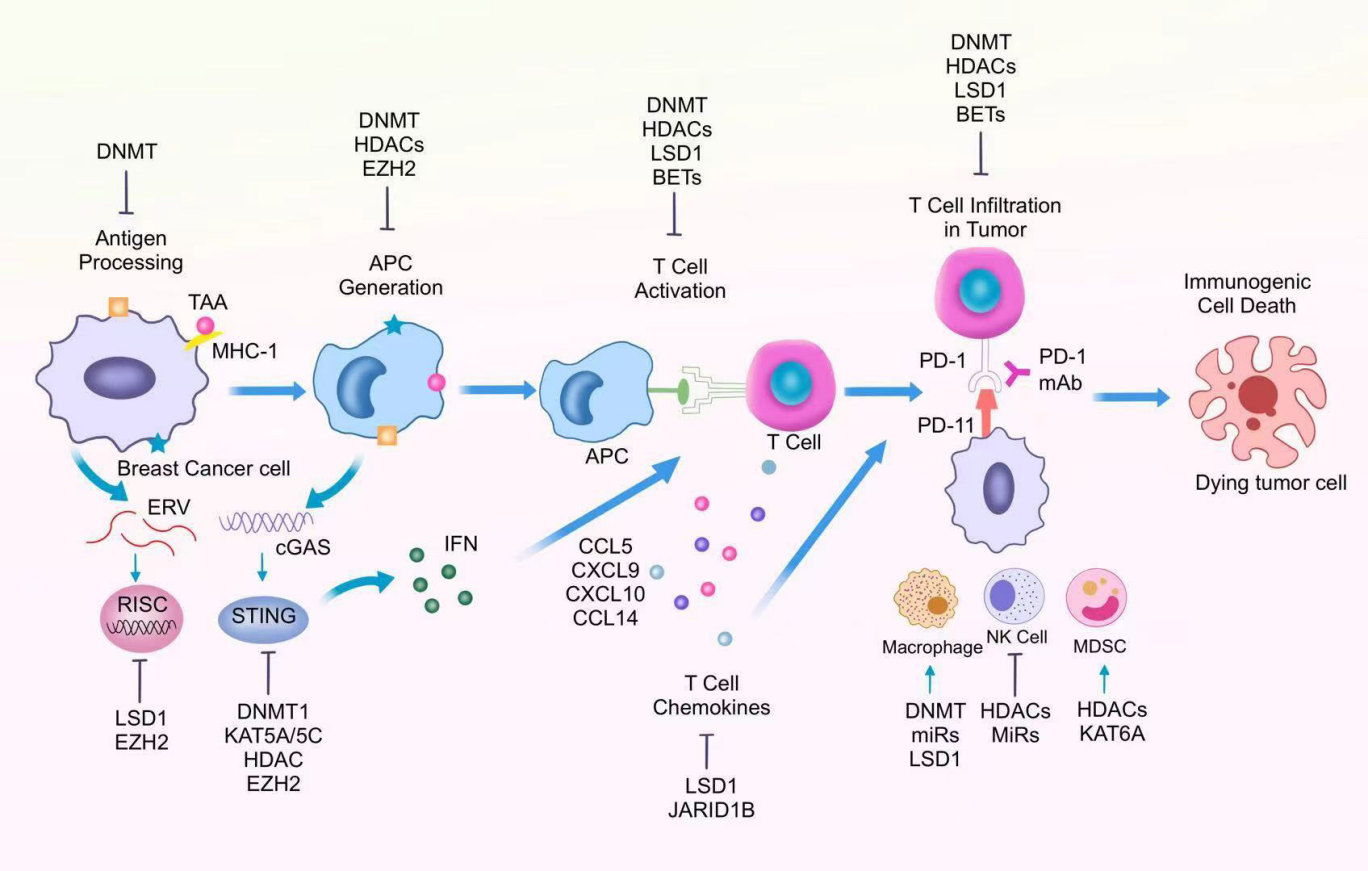
Epigenetic regulation of different immune cells in the tumor immunosuppressive microenvironment.

The normal anti-tumor immune response mainly consists of recognition by the immune system, and attack by the immune system to kill tumor cells. This process can also be referred to as the cancer immune cycle, which consists of several steps, including the generation and presentation process of tumor antigens, the activation process of immune cells, and the recognition and clearance of tumor cells (64). However, in BC, immune escape is one of the main characteristics of tumor cells. Recent studies have found that epigenetic dysregulation is the key to regulating tumor immune escape (**Figure 1**).

### 4.1 Generation of tumor antigens

Epigenetics can promote the formation of some neoantigens and abnormal antigen presentation processes during the generation and presentation of tumor antigens. CTA is a gene that is normally expressed only in male germ cells. Under normal conditions, CTA expression is precisely regulated by epigenetic mechanisms and is barely expressed in other cells (65). This allows the male testes to avoid the influence of immune cells. Recent studies have found that in BC, CTA-associated CpG sites are demethylated, an aberrant epigenetic mechanism that promotes CTA re-expression in tumors and leads to the formation of tumor neoantigens. DNA Methyltransferase Inhibitors Reactivate the CTA gene and promote tumor antigen generation, thereby enhancing anti-tumor immune responses (66).

### 4.2 Tumor antigen presentation

During antigen presentation, antigen-presenting cells display antigenic peptide fragments on MHC molecules on their surface. Through this process, T cells are effectively activated, which in turn initiates an adaptive immune response (67). In BC, MHC-I molecules are effectively inhibited, which in turn promotes immune escape of tumor cells. It has been found that in BC, the MHC-I gene is aberrantly methylated, resulting in MHC-I deletion, which in turn promotes dysregulated antigen presentation (67, 68). The use of DNA methyltransferase inhibitors (e.g., guadecitabine) can effectively reverse this process and promote MHC-I expression, thereby enhancing the anti-tumor immune response (69).

### 4.3 Activation of immune cells and the process of recognizing and eliminating tumor cells

Tumor-associated antigens (TAAs) expressed on the surface of tumor cells are processed into short peptide fragments and presented to the cell surface via MHC class I molecules.CD8+ T cells specifically recognize the MHC I-antigenic peptide complexes via the TCR, while the CD8 co-receptor enhances the binding stability. This process described above simultaneously requires strong activation signals from the dendritic cell pre-existing.

During the immune response, chemokines can recruit CD8^+^toxic T lymphocytes (CTLs) toward the tumor site. CTLs destroy cancer cells by secreting perforin, granzyme, and death ligands to induce an intrinsic apoptotic response in the cell. Increased expression of chemokines such as CCL5 and CXCL9 effectively promotes anti-tumor immune function of CTLs. In TNBC, overexpression of LSD1 was negatively correlated with the levels of chemokines that attract CD8^+^ T cells, and the expression of these chemokines could be repromoted by inhibiting LSD1 (70). Among other things, inhibition of LSD1 was closely associated with increased levels of H3K4me2 in the promoter region of the relevant chemokine genes. Thus, the use of LSD1 inhibitors enhances the migration of CD8^+^ T cells, which in turn enhances immunotherapy efficacy. In addition, the JmjC demethylase JARID1B bound to the LSD1/NuRD complex inhibited BC cell angiogenesis and metastasis by inhibiting the chemokine CCL14, which promotes immune cell activation (71). In BC cells, the demethylase Fbxl10 is recruited to the CCL7 promoter region and inhibits CCL7 expression and normal function. As an H3K4me3-targeted histone demethylase, knockdown of Fbxl10 promotes CCL7 expression, which in turn enhances anti-tumor immune responses (72). Targeting aberrant chemokines, in combination with immunotherapy, can enhance anti-tumor immunity and thus provide clinical benefit to BC patients (**Figure 1**).

## 5 Epigenetic modifications induce immunotherapy resistance in BC

Epigenetic modifications promote immunotherapy resistance in BC through multilevel mechanisms of action. Epigenetic modifications affect antigen presentation and inhibit anti-tumor immune cell function by regulating the expression of relevant genes, thereby impairing the anti-tumor immune response (73, 74). through aberrant methylation of DNA methyltransferase (DNMT1) and histone methyltransferase (EZH2), they inhibit the expression of MHC-I molecules The above processes lead to a decrease in antigen-presenting capacity on the surface of tumor cells, which in turn hinders the recognition and clearance of tumor cells by CD8+ T cells (75, 76). In addition, histone demethylase LSD1 reduces the infiltration of CD8+ T cells into the TME by inhibiting the expression of chemokines such as CCL5 and CXCL9, further weakening the immunotherapeutic response (77, 78). Notably, EZH2 also inhibits the expression of endogenous retroviruses (ERVs) through H3K27me3 deposition, blocks the activation of the cGAS-STING signaling pathway, and inhibits the production of type I interferon and its downstream T-cell initiation process, thereby enhancing the immune escape ability of tumor cells (79, 80). Together, these alterations in epigenetic modifications constitute an important molecular basis for BC immunotherapy resistance.

Furthermore, epigenetic modifications further exacerbate immunotherapy resistance by remodeling metabolic reprogramming pathways and promoting the formation of an immunosuppressive microenvironment. Histone demethylase KDM5B significantly suppresses innate immune responses by inhibiting the expression of STING, and consequently, the innate immune response (81). Moreover, it promotes tumor cell migration by regulating lipid metabolism. It was found that the tight binding of JARID1B to the LSD1/NuRD complex maintained an immunosuppressive microenvironment by inhibiting the expression of the chemokine CCL14 and limiting the activation of immune cells (71, 82). In addition, Fbxl10, an H3K4me3-targeted histone demethylase, is actively recruited to the CCL7 promoter region in BC and significantly inhibits its expression, ultimately inducing therapeutic resistance (72). Against the above targets, LSD1 inhibitors are able to restore chemokine function and enhance CD8+ T cell recruitment and activity. In contrast, KDM5 inhibitors can overcome BC resistance to trastuzumab by upregulating STING expression and reactivating the cGAS-STING signaling pathway (83). Wang et al. showed that HIF-1α transcriptionally activates the m6A demethylase FTO under hypoxic conditions, which in turn hinders the recognition and degradation of PDK1 by YTHDF3 by inhibiting the m6A-modified water of PDK1 mRNA. Further studies revealed that high expression of PDK1 activates the AKT/STAT3 signaling pathway, which ultimately stabilizes and upregulates PD-L1 expression and inhibits T cell activity (84). RPN1 (ribonucleoprotein I), a key regulator of membrane-bound glycosylation, is abnormally highly expressed in TNBC.RPN1 enhances the stability of PD-L1 by promoting its glycosylation modification, which mediates tumor cell immune escape. Knockdown of RPN1 could remodel TME and enhance the response rate to anti-PD-1 immunotherapy. The transcription factor YY1 regulates the expression of RPN1 by directly binding to its promoter region, whereas RPN1 and YBX1 synergize to form a regulatory axis, which together promote PD-L1 stability and high expression, and ultimately act as a resistance to immunotherapy (85, 86).

## 6 New strategies for epigenetics in BC treatment

### 6.1 Inhibitors of epigenetic modifying enzymes

Epigenetic modifying enzyme inhibitors have demonstrated significant potential in BC treatment by reversing abnormal epigenetic modifications and restoring normal gene expression patterns. These inhibitors mainly target DNA methyltransferases (DNMTs) and histone deacetylases (HDACs), effectively altering gene activity to inhibit tumor growth and progression.

#### 6.1.1 DNMTi

DNA methyltransferase inhibitors, such as Azacitidine and Decitabine, bind to DNMTs, preventing their methylation activity on DNA, thereby avoiding the methylation-induced silencing of tumor suppressor genes. These drugs restore the expression of tumor suppressor genes through demethylation, inhibiting tumor spread. For example, Decitabine can reactivate the silenced BRCA1 gene in BC, restoring its DNA repair function and enhancing the sensitivity of BC cells to chemotherapy (31). Decitabine and Eugenol, DNMTs inhibitors, can suppress the invasion and pro-angiogenic abilities of CAFs in breast cancer (BC), significantly improving the quality of life for patients.

#### 6.1.2 HDACi

Histone deacetylase inhibitors, such as Vorinostat and Panobinostat, inhibit the activity of HDACs, increasing the acetylation levels of histones, which leads to a more relaxed chromatin structure. When chromatin is more relaxed, it makes it easier for transcription factors to reach the DNA. This helps turn on genes that can stop tumors. In BC, drugs that block HDAC can slow down cell growth and cause cells to die by turning on important genes like p21 (26, 33). Recent studies suggest that epigenetic modifying enzyme inhibitors can be used not only as monotherapies but also in combination with other treatments to enhance therapeutic efficacy. The combination of HDAC inhibitors with ICIs has shown stronger anti-tumor activity, potentially overcoming resistance in certain BC (33, 34). HDAC inhibitors like Scriptaid and ACY1215 can inhibit the activity of CAFs and the aggregation of M2 macrophages, while promoting the recruitment of CD8 T cells (87).

### 6.2 Therapy of non-coding RNAs

#### 6.2.1 Mechanism and impact of miRNA mimics

**Figure 2:**
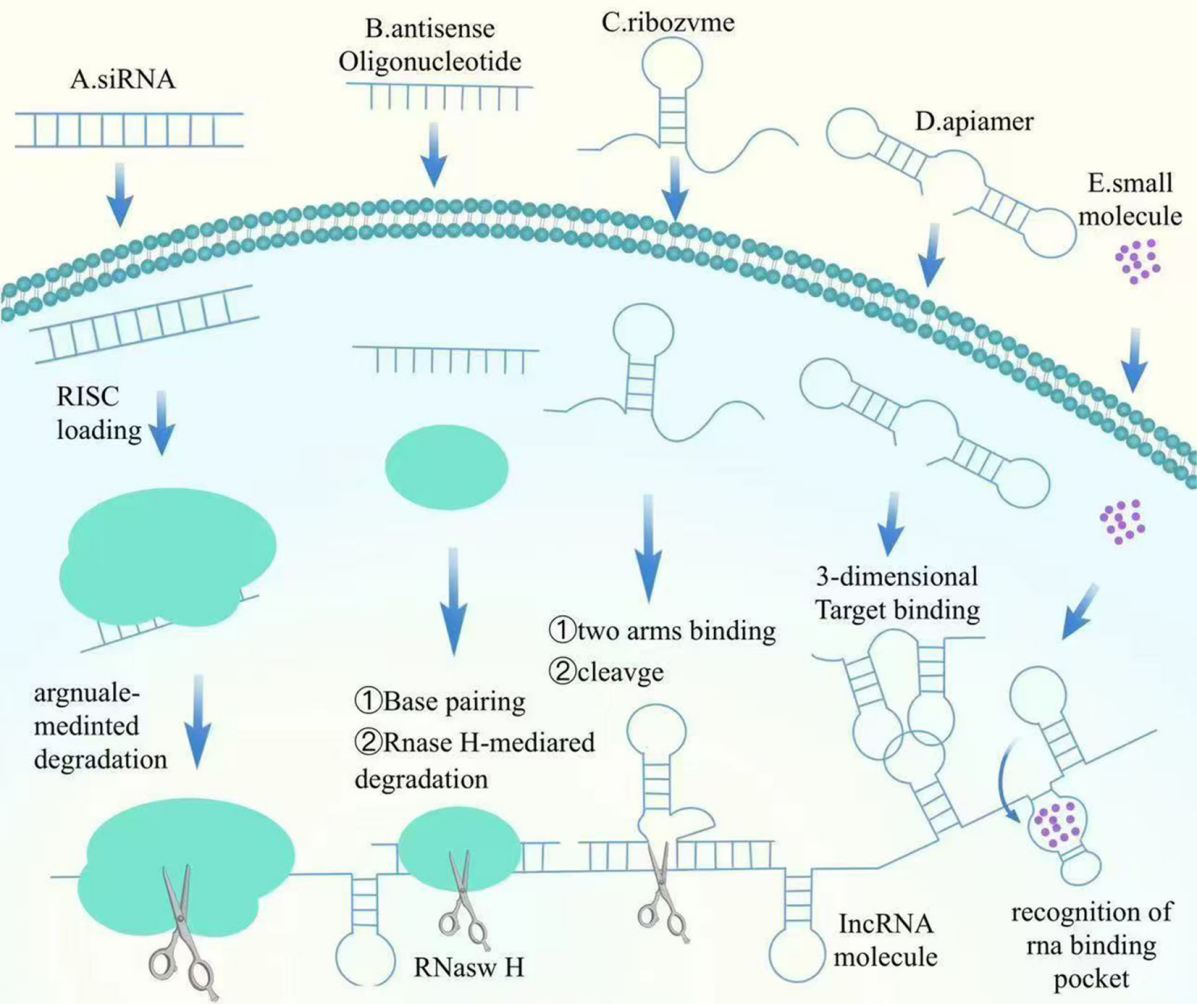
Mechanisms of action of targeted lncRNA factors.

NcRNAs can closely play biological roles through various epigenetic modifying enzymes with DAN methylation, histone modification, etc., which in turn regulate the expression of related genes and ultimately regulate the proliferation and metastasis of BC. miRNA mimics are synthetic miRNA molecules designed to restore or enhance the function of specific miRNAs, thus regulating gene expression. In BC, the expression of certain tumor-suppressive miRNAs, such as miR-34a and miR-15/16, is often downregulated. Introducing miRNA mimics can restore the levels of these tumor-suppressive miRNAs, thereby inhibiting oncogene expression and inducing apoptosis in tumor cells. As an example, miR-34a mimics have actually demonstrated significant anti-tumor results in preclinical research studies, successfully preventing the expansion and movement of BC cells (31). The lncRNA MIAT promotes DLG3 methylation and expression down-regulation by recruiting DNMTs to the DLG3 promoter region. And silencing of MIAT restored DLG3 levels while significantly activating the expression of the Hippo signaling pathway, ultimately inhibiting BC growth and proliferation (88). The expression of circMETTL3 is mainly dependent on METTL3-mediated m6A modification, and its expression is significantly decreased upon METTL3 knockdown. It was found that METTL3 knockdown was also accompanied by a significant decrease in m6A modification. circMETTL3 indirectly promotes tumorigenesis by competitively binding to miR-31-5p and deregulating the inhibition of target gene CDK1 by this microRNA. And METTL3 deficiency can weaken this oncogenic axis by downregulating circMETTL3 (89). Here, we summarize the mechanism of action of the targeted lncRNA factors (**Figure 2**).

#### 6.2.2 Mechanism and impact of antisense oligonucleotides

ASOs are short single-stranded DNA or RNA oligonucleotides that hybridize with target mRNA, avoiding its translation or inducing its degradation. By especially binding to oncogene mRNAs or other cancer-related mRNAs, ASOs inhibit their expression, therefore halting lump progression. The BCL-2 gene, which inscribes a healthy protein that helps cancer cells withstand apoptosis, is typically overexpressed in BC. ASOs targeting BCL-2 mRNA can minimize its expression, bring back the apoptotic ability of cells. In BC versions, ASOs targeting BCL-2 have shown considerable anti-tumor effects (33). Additionally, in hormone-dependent BC, the high expression of estrogen receptor alpha (ERα) advertises lump growth. ASOs can substantially reduce ERα protein degrees by inhibiting the translation of ERα mRNA, thereby inhibiting growth cell spreading (26). Current research studies recommend that ASOs hold terrific possible in BC treatment, especially in personalized therapies targeting specific gene expressions. With innovations in delivery systems and enhanced targeting, ASOs are anticipated to be used in mix with various other treatments to increase precision and effectiveness. Study is likewise exploring the combination of ASOs with immunotherapy to further enhance their anti-tumor results (90).

## 7 Targeted epigenetics and immunotherapy

In BC treatment, combining epigenetic treatments with immunotherapy has demonstrated substantial synergistic impacts. Epigenetic therapies modify cancer cell gene expression, improving the body immune system’s capacity to recognize and strike growths. Concurrently, immunotherapy enhances the individual’s immune response, amplifying the general anti-cancer result.

### 7.1 Targeting aberrant DNA methylation to enhance immunotherapy effect

Epigenetic therapies, such as DNMTi and HDACi, can reverse abnormal epigenetic alterations in lump cells, reactivating silenced antigens and immune-related genetics. This reactivation enhances the presence of tumor antigens, facilitating immune cell recognition and assault on tumors. As an example, HDAC inhibitors can lower PD-L1 expression, making tumors extra at risk to immune checkpoint inhibitors, therefore enhancing the effectiveness of immunotherapy (91–93). The combination of DNMTi targeting and immunotherapy is of significant importance in BC treatment. Research has shown that monotherapy with anti-PD-1 is ineffective in halting tumor progression in some BC patients (94). However, the combination of DNMTi with anti-PD-1 inhibitors significantly improves patient survival rates and quality of life, with tumor burden reduced by over 80% (95). Additionally, a phase II clinical trial found that the DNA demethylating agent CC-486, when combined with the anti-PD-1 antibody durvalumab, achieved excellent therapeutic outcomes in metastatic BC patients and was very safe for the patients (96).

### 7.2 Combination of histone modifying enzyme inhibitors with immunotherapy

HDACi can improve the response to PD-1 inhibitors by upregulating the expression of PD-L1 and HLA-DR and inhibiting Treg activity. Moreover, the combination of HDACi and ICIs can promote the infiltration and function of anti-tumor immune cells, suppress the activity of MDSCs and immune-resistant breast tumor cells, thereby enhancing a stronger anti-tumor immune response (97–99). EZH2, a histone methyltransferase, promotes gene silencing through H3K27me3. In BC, overexpression of EZH2 is connected to lump aggressiveness and immune evasion. Preventing EZH2 can protect against H3K27me3 development, lifting the suppression of immune-related genetics and enhancing immune cell attack on growths. Research studies show that incorporating EZH2 inhibitors with anti-PD-1/PD-L1 therapies significantly improves BC treatment results and decreases tumor worry (93, 100). Kim and colleagues demonstrated that the combination of entinostat and anti-CTLA-4 antibody was effective in inhibiting MDSC activity, thereby significantly inhibiting BC metastasis. Phase I clinical trial finds entinostat and nivolumab achieved significant therapeutic efficacy in treating metastatic HER2-negative BC patients (101, 102). The trial confirmed that the patients showed good safety and tolerance, with a low incidence of immune-related adverse events, and a significant improvement in the patients’ quality of life. Here, we summarize relevant clinical trials combining targeted epigenetic agents and immunotherapy (**Table 2**).

**Table 2:** The relevant clinical trials combining targeted epigenetic agents and immunotherapy.

NCT Number	Study Title	Study Status	Conditions	Interventions	Phases
NCT02395627	Reversing Therapy Resistance With Epigenetic-Immune Modification	TERMINATED	Breast Neoplasms	DRUG: Tamoxifen|DRUG: Vorinostat|DRUG: Pembrolizumab	PHASE2
NCT04190056	Pembrolizumab and Tamoxifen With or Without Vorinostat for the Treatment of Estrogen Receptor Positive Breast Cancer	TERMINATED	Anatomic Stage IV Breast Cancer AJCC v8|Prognostic Stage IV Breast Cancer AJCC v8	BIOLOGICAL: Pembrolizumab|DRUG: Tamoxifen|DRUG: Vorinostat	PHASE2
NCT05680662	The Study of Quadruple Therapy Quercetin, Zinc, Metformin, and EGCG as Adjuvant Therapy for Early, Metastatic Breast Cancer and Triple-negative Breast Cancer, a Novel Mechanism	UNKNOWN	Breast Cancer Female|Triple Negative Breast Cancer	COMBINATION_PRODUCT: quercetin, EGCG, metformin, zinc	EARLY_PHASE1
NCT04335669	NordicTrip, a Translational Study of Preoperative Chemotherapy in TNBC	RECRUITING	Breast Cancer|Triple Negative Breast Neoplasms	DRUG: epirubicin, cyclophosphamide, paclitaxel, carboplatin, pembrolizumab|DRUG: epirubicin, cyclophosphamide, capecitabine, paclitaxel, carboplatin, pembrolizumab	PHASE3

## 8 Limitations and future outlook

### 8.1 Limitations

Targeting epigenetic modification targets currently shows great potential in BC therapy, but still faces many challenges. First, epigenetic drugs have low specificity and selectivity. This can make them interfere with some specific genes in a way that may affect the expression of other normal genes, leading to side effects. Moreover, some BC patients may develop resistance to epigenetic drugs or even escape drug treatment through reprogramming (74). Second, the mechanism of action of epigenetic modifications in different cell types and microenvironments is extremely complex, which greatly increases the difficulty of targeted therapy (103). Existing drugs and technologies are difficult to effectively regulate multiple epigenetic pathways or modifications at the same time, and it is difficult to comprehensively restore the normal pattern of gene expression (104); therefore, optimal efficacy may not be achieved through intervention at a single target only. In addition, long-term interventions of epigenetic modifications may result in erratic effects or potential genomic alterations, and these uncertainties may lead to new health risks (105, 106). Finally, numerous challenges remain in the process of applying epigenetics to the clinic, including multiple stages of preclinical studies, clinical trials, and drug development.

### 8.2 Future outlook

As epigenetics research continues to deepen, especially in areas such as oncology and immunotherapy, the study of epigenetic modification targets has provided a completely new direction for the treatment of tumor patients (100). The biggest limitation of immunotherapy is that about 40% of patients do not respond to the treatment and the side effects are more significant. The combination of targeting epigenetic modifications and immunotherapy may be the key to promoting patient response to treatment in the future. In the future, personalized medicine is expected to achieve more individualized and efficient therapeutic strategies by targeting epigenetic modifications and precisely regulating gene expression. The combination of epigenetic drugs with traditional therapies such as immunotherapy may result in more effective combination therapies that increase the efficacy of treatment and reduce the development of drug resistance (107). Novel inhibitors of epigenetic modifications, such as those targeting DNMTs and HDACs (97, 108), will hopefully play a greater role in the clinic. Meanwhile, the application of epigenetics in regenerative medicine is promising. Finally, the discovery of epigenetic markers contributes to the early diagnosis and prognostic assessment of tumors, and they may become routine screening tools in the future, significantly improving the survival rate and quality of life of patients.

## 9 Conclusion

Epigenetics is critical in the onset, progression, immune escape, and treatment of BC. The development of BC is usually closely associated with aberrant epigenetic modifications. Notably, epigenetic modifications in turn tightly regulate immune cell proliferation, differentiation, and function. It also plays a major role in promoting immune escape from BC cells. Therapeutically, inhibitors targeting epigenetic modifying enzymes are able to reverse aberrant epigenetic changes and thus show significant anti-tumor activity. When used in combination with immunotherapy, these inhibitors can further improve efficacy and overall patient survival. In conclusion, epigenetics has provided new perspectives for a deeper understanding of the pathogenesis of BC and proposed new targets and strategies for its treatment.

## References

[1] Sung H Ferlay J Siegel RL Laversanne M Soerjomataram I Jemal A. Global cancer statistics 2020: GLOBOCAN estimates of incidence and mortality worldwide for 36 cancers in 185 countries. CA Cancer J Clin. (2021) 71:209–49. doi: 10.3322/caac.21660 33538338

[2] Bingfeng H Rongshou Z Hongmei Z Shaoming W Kexin S Ru C. Cancer incidence and mortality in China, 2022. J Natl Cancer Center. (2024) 4:47–53. doi: 10.1016/j.jncc.2024.01.006 39036382 PMC11256708

[3] Xiong X Zheng LW Ding Y Chen YF Cai YW Wang LP. Breast cancer: Pathogenesis and treatments. Signal Transduct Target Ther. (2025) 10:49. doi: 10.1038/s41392-024-02108-4 39966355 PMC11836418

[4] Valente S Roesch E. Breast cancer survivorship. J Surg Oncol. (2024) 130:8–15. doi: 10.1002/jso.v130.1 38534002

[5] Ji YT Liu SW Zhang YM Duan HY Liu XM Feng ZW. Comparison of the latest cancer statistics, cancer epidemic trends and determinants between China and the United States. Zhonghua Zhong Liu Za Zhi. (2024) 46:1–11. doi: 10.3760/cma.j.cn112152-20240208-00068 38764329

[6] Xiu M Lu Y Wang X Fan Y Li Q Li Q. Dose-dense paclitaxel plus carboplatin in combination with trastuzumab neoadjuvant versus standard adjuvant therapy in human epidermal growth factor receptor-2 positive and hormone receptor negative breast cancer: a prospective cohort study. Zhonghua Zhong Liu Za Zhi. (2023) 45:709–16. doi: 10.3760/cma.j.cn112152-20221006-00678 37580278

[7] Yue J Song GH Li HP Sun T Song LH Tong ZS. Gemcitabine long-term maintenance chemotherapy benefits patients with survival: a multicenter, real-world study of advanced breast cancer treatment in China. Zhonghua Zhong Liu Za Zhi. (2024) 46:249–55. doi: 10.3760/cma.j.cn112152-20231024-00251 38494771

[8] Xu XS Ding H Zhang X Liao Y Li H Liu QY. Clinical characteristics and prognosis of patients with therapy-related myelodysplastic syndrome and acute myeloid leukemia arising from Malignant tumors. Zhonghua Xue Ye Xue Za Zhi. (2023) 44:742–8. doi: 10.3760/cma.j.issn.0253-2727.2023.09.007 PMC10630571 38049318

[9] Yadav BS Dahiya D Kannan P Goyal S Laroiya I Irrinki S. HYPofractionated Adjuvant RadioTherapy in 1 versus 2 weeks in high-risk patients with breast cancer (HYPART): a non-inferiority, open-label, phase III randomized trial. Trials. (2024) 25:21. doi: 10.1186/s13063-023-07851-7 38167339 PMC10763219

[10] Limninart N Harvey JA Schultz KJ Mills AM Noland MMB Schroen AT. What do you mean it’s not cancer?” A review of autoimmune and systemic inflammatory diseases involving the breast. J Breast Imaging. (2021) 3:612–25. doi: 10.1093/jbi/wbab029 38424941

[11] Ahmadpour ST Orre C Bertevello PS Mirebeau-Prunier D Dumas JF Desquiret-Dumas V. Breast Cancer Chemoresistance: Insights into the Regulatory Role of lncRNA. Int J Mol Sci. (2023) 24:15897. doi: 10.3390/ijms242115897 37958880 PMC10650504

[12] Biswas S Rao CM. Epigenetics in cancer: Fundamentals and beyond. Pharmacol Ther. (2017) 173:118–34. doi: 10.1016/j.pharmthera.2017.02.011 28188812

[13] Wong CC Qian Y Yu J. Interplay between epigenetics and metabolism in oncogenesis: mechanisms and therapeutic approaches. Oncogene. (2017) 36:3359–74. doi: 10.1038/onc.2016.485 PMC5485177 28092669

[14] Shi J Li YM Fang XD. The mechanism and clinical significance of long noncoding RNA-mediated gene expression via nuclear architecture. Yi Chuan. (2017) 39:189–99. doi: 10.16288/j.yczz.16-385 28420615

[15] Guo Z Saw PE Jon S. Non-invasive physical stimulation to modulate the tumor microenvironment: Unveiling a new frontier in cancer therapy. Bio Integr. (2024) 5:1–14. doi: 10.15212/bioi-2024-0012

[16] Shuai Y Ma Z Ju J Wei T Gao S Kang Y. Liquid-based biomarkers in breast cancer: Looking beyond the blood. J Transl Med. (2023) 21:809. doi: 10.1186/s12967-023-04660-z 37957623 PMC10644618

[17] Li M Li L Zheng J Li Z Li S Wang K. Liquid biopsy at the frontier in renal cell carcinoma: Recent analysis of techniques and clinical application. Mol Cancer. (2023) 22:37. doi: 10.1186/s12943-023-01745-7 36810071 PMC9942319

[18] Dai E Zhu Z Wahed S Qu Z Storkus WJ Guo ZS. Epigenetic modulation of antitumor immunity for improved cancer immunotherapy. Mol Cancer. (2021) 20:171. doi: 10.1186/s12943-021-01464-x 34930302 PMC8691037

[19] Agarwal G Kudapa H Ramalingam A Choudhary D Sinha P Garg V. Epigenetics and epigenomics: Underlying mechanisms, relevance, and implications in crop improvement. Funct Integr Genomics. (2020) 20:739–61. doi: 10.1007/s10142-020-00756-7 33089419

[20] Jang HS Shin WJ Lee JE Do JT. CpG and non-cpG methylation in epigenetic gene regulation and brain function. Genes (Basel). (2017) 8:148. doi: 10.3390/genes8060148 28545252 PMC5485512

[21] Lawrence M Daujat S Schneider R. Lateral thinking: How histone modifications regulate gene expression. Trends Genet. (2016) 32:42–56. doi: 10.1016/j.tig.2015.10.007 26704082

[22] Jiang D Li T Guo C Tang TS Liu H. Small molecule modulators of chromatin remodeling: From neurodevelopment to neurodegeneration. Cell Biosci. (2023) 13:10. doi: 10.1186/s13578-023-00953-4 36647159 PMC9841685

[23] Ratti M Lampis A Ghidini M Salati M Mirchev MB Valeri N. MicroRNAs (miRNAs) and long non-coding RNAs (lncRNAs) as new tools for cancer therapy: First steps from bench to bedside. Target Oncol. (2020) 15:261–78. doi: 10.1007/s11523-020-00717-x PMC7283209 32451752

[24] Yan Y Su L Huang S He Q Lu J Luo H. Circadian rhythms and breast cancer: Unraveling the biological clock’s role in tumor microenvironment and ageing. Front Immunol. (2024) 15:1444426. doi: 10.3389/fimmu.2024.1444426 39139571 PMC11319165

[25] Zhang L Lu Q Chang C. Epigenetics in health and disease. Adv Exp Med Biol. (2020) 1253:3–55. doi: 10.1007/978-981-15-3449-2_1 32445090

[26] Castro-Muñoz LJ Ulloa EV Sahlgren C Lizano M de la Cruz-Hernández E Contreras-Paredes A. Modulating epigenetic modifications for cancer therapy (Review). Oncol Rep. (2023) 49:59. doi: 10.3892/or.2023.8496 36799181 PMC9942256

[27] Ji X Tian X Feng S Zhang L Wang J Guo R. Intermittent F-actin perturbations by magnetic fields inhibit breast cancer metastasis. Res (Washington DC). (2023) 6:0080. doi: 10.34133/research.0080 PMC10017101 36939445

[28] Tang J He J Guo H Lin H Li M Yang T. PTBP2-mediated alternative splicing of IRF9 controls tumor-associated monocyte/macrophage chemotaxis and repolarization in neuroblastoma progression. Res (Washington DC). (2023) 6:0033. doi: 10.34133/research.0033 PMC10076020 37040518

[29] Guo XF Gu SS Wang J Sun H Zhang YJ Yu PF. Protective effect of mesenchymal stem cell-derived exosomal treatment of hippocampal neurons against oxygen-glucose deprivation/reperfusion-induced injury. World J Emergency Med. (2022) 13:46–53. doi: 10.5847/wjem.j.1920-8642.2022.015 PMC8677914 35003415

[30] Luo Z Mei J Wang X Wang R He Z Geffen Y. Voluntary exercise sensitizes cancer immunotherapy via the collagen inhibition-orchestrated inflammatory tumor immune microenvironment. Cell Rep. (2024) 43:114697. doi: 10.1016/j.celrep.2024.114697 39217611

[31] Costa P Sales SLA Pinheiro DP Pontes LQ Maranhão SS Pessoa C. Epigenetic reprogramming in cancer: From diagnosis to treatment. Front Cell Dev Biol. (2023) 11:1116805. doi: 10.3389/fcell.2023.1116805 36866275 PMC9974167

[32] Barrett JE Herzog C Jones A Leavy OC Evans I Knapp S. The WID-BC-index identifies women with primary poor prognostic breast cancer based on DNA methylation in cervical samples. Nat Commun. (2022) 13:449. doi: 10.1038/s41467-021-27918-w 35105882 PMC8807602

[33] Falahi F van Kruchten M Martinet N Hospers GA Rots MG. Current and upcoming approaches to exploit the reversibility of epigenetic mutations in breast cancer. Breast Cancer Res. (2014) 16:412. doi: 10.1186/s13058-014-0412-z 25410383 PMC4303227

[34] Chong SY Cutler S Lin J-J Tsai C-H Tsai H-K Biggins S. H3K4 methylation at active genes mitigates transcription-replication conflicts during replication stress. Nat Commun. (2020) 11:809. doi: 10.1038/s41467-020-14595-4 32041946 PMC7010754

[35] Tang G Xia H Huang Y Guo Y Chen Y Ma Z. Liquid–liquid phase separation of H3K27me3 reader BP1 regulates transcriptional repression. Genome Biol. (2024) 25:67. doi: 10.1186/s13059-024-03209-7 38468348 PMC10926671

[36] Di L Zhu W-G. The role of H3K27me3 methylation in cancer development. Genome Instability Dis. (2024) 5:17–34. doi: 10.1007/s42764-023-00118-0

[37] Qiao W Liu H Liu R Liu Q Zhang T Guo W. Prognostic and clinical significance of histone deacetylase 1 expression in breast cancer: A meta-analysis. Clin Chim Acta. (2018) 483:209–15. doi: 10.1016/j.cca.2018.05.005 29738697

[38] Chen C Liu J. Histone acetylation modifications: A potential targets for the diagnosis and treatment of papillary thyroid cancer. Front Oncol. (2022) 12:1053618. doi: 10.3389/fonc.2022.1053618 36523971 PMC9745171

[39] Wang X Xu J Sun Y Cao S Zeng H Jin N. Hedgehog pathway orchestrates the interplay of histone modifications and tailors combination epigenetic therapies in breast cancer. Acta Pharm Sin B. (2023) 13:2601–12. doi: 10.1016/j.apsb.2023.03.009 PMC10326305 37425067

[40] Liu S Chang W Jin Y Feng C Wu S He J. The function of histone acetylation in cervical cancer development. Biosci Rep. (2019) 39:BSR20190527. doi: 10.1042/bsr20190527 30886064 PMC6465204

[41] Wang F Zhang W Song Z Wang M Wu H Yang Y. A novel miRNA inhibits metastasis of prostate cancer via decreasing CREBBP-mediated histone acetylation. J Cancer Res Clin Oncol. (2021) 147:469–80. doi: 10.1007/s00432-020-03455-9 PMC11802035 33221996

[42] Sarkar T Dhar S Chakraborty D Pati S Bose S Panda AK. FOXP3/HAT1 axis controls treg infiltration in the tumor microenvironment by inducing CCR4 expression in breast cancer. Front Immunol. (2022) 13:740588. doi: 10.3389/fimmu.2022.740588 35222362 PMC8863663

[43] Gupta A Guerin-Peyrou TG Sharma GG Park C Agarwal M Ganju RK. The mammalian ortholog of Drosophila MOF that acetylates histone H4 lysine 16 is essential for embryogenesis and oncogenesis. Mol Cell Biol. (2008) 28:397–409. doi: 10.1128/MCB.01045-07 17967868 PMC2223300

[44] Singh M Bacolla A Chaudhary S Hunt CR Pandita S Chauhan R. Histone acetyltransferase MOF orchestrates outcomes at the crossroad of oncogenesis, DNA damage response, proliferation, and stem cell development. Mol Cell Biol. (2020) 40:e00232-20. doi: 10.1128/MCB.00232-20 32661120 PMC7459263

[45] Ma L Qi L Li S Yin Q Liu J Wang J. Aberrant HDAC3 expression correlates with brain metastasis in breast cancer patients. Thorac Cancer. (2020) 11:2493–505. doi: 10.1111/1759-7714.13561 PMC7471029 32686908

[46] Garmpis N Damaskos C Dimitroulis D Kouraklis G Garmpi A Sarantis P. Clinical significance of the histone deacetylase 2 (HDAC-2) expression in human breast cancer. J Pers Med. (2022) 12:1672. doi: 10.3390/jpm12101672 36294811 PMC9604828

[47] Zhao Y He J Yang L Luo Q Liu Z. Histone deacetylase-3 modification of microRNA-31 promotes cell proliferation and aerobic glycolysis in breast cancer and is predictive of poor prognosis. J Breast Cancer. (2018) 21:112–23. doi: 10.4048/jbc.2018.21.2.112 PMC6015978 29963106

[48] Wei H Ma W Lu X Liu H Lin K Wang Y. KDELR2 promotes breast cancer proliferation via HDAC3-mediated cell cycle progression. Cancer Commun (Lond). (2021) 41:904–20. doi: 10.1002/cac2.12180 PMC8441056 34146461

[49] Andreani C Bartolacci C Persico G Casciaro F Amatori S Fanelli M. SIRT6 promotes metastasis and relapse in HER2-positive breast cancer. Sci Rep. (2023) 13:22000. doi: 10.1038/s41598-023-49199-7 38081972 PMC10713583

[50] Agelaki S Papadaki C Stoupis I Tsalikis L Papadaki MA Monastirioti A. Role of circulating miRNAs in detecting metastasis and having prognostic significance in metastatic breast cancer. J Clin Oncol. (2018) 36:1086–6. doi: 10.1200/JCO.2018.36.15_suppl.1086

[51] Wang Y Wu S Zhu X Zhang L Deng J Li F. LncRNA-encoded polypeptide ASRPS inhibits triple-negative breast cancer angiogenesis. J Exp Med. (2020) 217:jem.20190950. doi: 10.1084/jem.20190950 31816634 PMC7062514

[52] Guo B Wu S Zhu X Zhang L Deng J Li F. Micropeptide CIP2A-BP encoded by LINC00665 inhibits triple-negative breast cancer progression. EMBO J. (2020) 39:e102190. doi: 10.15252/embj.2019102190 31755573 PMC6939193

[53] Xiu B Chi Y Liu L Chi W Zhang Q Chen J. LINC02273 drives breast cancer metastasis by epigenetically increasing AGR2 transcription. Mol Cancer. (2019) 18:187. doi: 10.1186/s12943-019-1115-y 31856843 PMC6921600

[54] Xiong H Shen J Chen Z Yang J Xie B Jia Y. H19/let−7/Lin28 ceRNA network mediates autophagy inhibiting epithelial−mesenchymal transition in breast cancer. Int J Oncol. (2020) 56:794–806. doi: 10.3892/ijo.2020.4967 32124962

[55] Ni C Fang QQ Chen WZ Jiang JX Jiang Z Ye J. Breast cancer-derived exosomes transmit lncRNA SNHG16 to induce CD73+γδ1 Treg cells. Signal Transduct Target Ther. (2020) 5:41. doi: 10.1038/s41392-020-0129-7 32345959 PMC7188864

[56] Zhang S Ma F Xie X Shen Y. Prognostic value of long non-coding RNAs in triple negative breast cancer: A PRISMA-compliant meta-analysis. Med (Baltimore). (2020) 99:e21861. doi: 10.1097/MD.0000000000021861 PMC7489686 32925722

[57] Takada M Toi M. Neoadjuvant treatment for HER2-positive breast cancer. Chin Clin Oncol. (2020) 9:32. doi: 10.21037/cco-20-123 32527117

[58] Zhong G Wang K Li J Xiao S Wei W Liu J. Determination of serum exosomal H19 as a noninvasive biomarker for breast cancer diagnosis. Onco Targets Ther. (2020) 13:2563–71. doi: 10.2147/OTT.S243601 PMC7109356 32273726

[59] Arun G Aggarwal D Spector DL. MALAT1 long non-coding RNA: Functional implications. Noncoding RNA. (2020) 6:22. doi: 10.3390/ncrna6020022 32503170 PMC7344863

[60] Goyal B Yadav SRM Awasthee N Gupta S Kunnumakkara AB Gupta SC. Diagnostic, prognostic, and therapeutic significance of long non-coding RNA MALAT1 in cancer. Biochim Biophys Acta Rev Cancer. (2021) 1875:188502. doi: 10.1016/j.bbcan.2021.188502 33428963

[61] Vishnubalaji R Alajez NM. Epigenetic regulation of triple negative breast cancer (TNBC) by TGF-β signaling. Sci Rep. (2021) 11:15410. doi: 10.1038/s41598-021-94514-9 34326372 PMC8322425

[62] Zhang X Zhuang J Liu L He Z Liu C Ma X. Integrative transcriptome data mining for identification of core lncRNAs in breast cancer. PeerJ. (2019) 7:e7821. doi: 10.7717/peerj.7821 31608179 PMC6786248

[63] Tian T Gong Z Wang M Hao R Lin S Liu K. Identification of long non-coding RNA signatures in triple-negative breast cancer. Cancer Cell Int. (2018) 18:103. doi: 10.1186/s12935-018-0598-8 30026672 PMC6050698

[64] Cao J Yan Q. Cancer epigenetics, tumor immunity, and immunotherapy. Trends Cancer. (2020) 6:580–92. doi: 10.1016/j.trecan.2020.02.003 PMC7330177 32610068

[65] Li Y Li J Wang Y Zhang Y Chu J Sun C. Roles of cancer/testis antigens (CTAs) in breast cancer. Cancer Lett. (2017) 399:64–73. doi: 10.1016/j.canlet.2017.02.031 28274891

[66] Jakobsen MK Traynor S Stæhr M Duijf PG Nielsen AY Terp MG. The cancer/testis antigen gene VCX2 is rarely expressed in Malignancies but can be epigenetically activated using DNA methyltransferase and histone deacetylase inhibitors. Front Oncol. (2020) 10:584024. doi: 10.3389/fonc.2020.584024 33634013 PMC7900521

[67] DhatChinamoorthy K Colbert JD Rock KL. Cancer immune evasion through loss of MHC class I antigen presentation. Front Immunol. (2021) 12:636568. doi: 10.3389/fimmu.2021.636568 33767702 PMC7986854

[68] Taylor BC Balko JM. Mechanisms of MHC-I downregulation and role in immunotherapy response. Front Immunol. (2022) 13:844866. doi: 10.3389/fimmu.2022.844866 35296095 PMC8920040

[69] Luo N Nixon MJ Gonzalez-Ericsson PI Sanchez V Opalenik SR Li H. DNA methyltransferase inhibition upregulates MHC-I to potentiate cytotoxic T lymphocyte responses in breast cancer. Nat Commun. (2018) 9:248. doi: 10.1038/s41467-017-02630-w 29339738 PMC5770411

[70] Qin Y Vasilatos SN Chen L Wu H Cao Z Fu Y. Inhibition of histone lysine-specific demethylase 1 elicits breast tumor immunity and enhances antitumor efficacy of immune checkpoint blockade. Oncogene. (2019) 38:390–405. doi: 10.1038/s41388-018-0451-5 30111819 PMC6336685

[71] Li Q Shi L Gui B Yu W Wang J Zhang D. Binding of the JmjC demethylase JARID1B to LSD1/NuRD suppresses angiogenesis and metastasis in breast cancer cells by repressing chemokine CCL14. Cancer Res. (2011) 71:6899–908. doi: 10.1158/0008-5472.can-11-1523 21937684

[72] Janzer A Stamm K Becker A Zimmer A Buettner R Kirfel J. The H3K4me3 histone demethylase Fbxl10 is a regulator of chemokine expression, cellular morphology, and the metabolome of fibroblasts. J Biol Chem. (2012) 287:30984–92. doi: 10.1074/jbc.m112.341040 PMC3438931 22825849

[73] Kundu M Butti R Panda VK Malhotra D Das S Mitra T. Modulation of the tumor microenvironment and mechanism of immunotherapy-based drug resistance in breast cancer. Mol Cancer. (2024) 23:92. doi: 10.1186/s12943-024-01990-4 38715072 PMC11075356

[74] Yin J Gu T Chaudhry N Davidson NE Huang Y. Epigenetic modulation of antitumor immunity and immunotherapy response in breast cancer: Biological mechanisms and clinical implications. Front Immunol. (2023) 14:1325615. doi: 10.3389/fimmu.2023.1325615 38268926 PMC10806158

[75] Zhang Y Chen J Liu H Mi R Huang R Li X. The role of histone methylase and demethylase in antitumor immunity: A new direction for immunotherapy. Front Immunol. (2022) 13:1099892. doi: 10.3389/fimmu.2022.1099892 36713412 PMC9874864

[76] Dunn J Rao S. Epigenetics and immunotherapy: The current state of play. Mol Immunol. (2017) 87:227–39. doi: 10.1016/j.molimm.2017.04.012 28511092

[77] Khodayari S Khodayari H Saeedi E Mahmoodzadeh H Sadrkhah A Nayernia K. Single-cell transcriptomics for unlocking personalized cancer immunotherapy: Toward targeting the origin of tumor development immunogenicity. Cancers. (2023) 15:3615. doi: 10.3390/cancers15143615 37509276 PMC10377122

[78] Lee DY Salahuddin T Iqbal J. Lysine-specific demethylase 1 (LSD1)-mediated epigenetic modification of immunogenicity and immunomodulatory effects in breast cancers. Curr Oncol (Toronto Ont). (2023) 30:2127–43. doi: 10.3390/curroncol30020164 PMC9955398 36826125

[79] Deblois G Tonekaboni SAM Grillo G Martinez C Kao YI Tai F. Epigenetic switch-Induced viral mimicry evasion in chemotherapy-Resistant breast cancer. Cancer Discov. (2020) 10:1312–29. doi: 10.1158/2159-8290.cd-19-1493 32546577

[80] Sheng W LaFleur MW Nguyen TH Chen S Chakravarthy A Conway JR. LSD1 ablation stimulates anti-tumor immunity and enables checkpoint blockade. Cell. (2018) 174:549–563.e519. doi: 10.1016/j.cell.2018.05.052 29937226 PMC6063761

[81] Zhang ZG Zhang HS Sun HL Liu HY Liu MY Zhou Z. KDM5B promotes breast cancer cell proliferation and migration via AMPK-mediated lipid metabolism reprogramming. Exp Cell Res. (2019) 379:182–90. doi: 10.1016/j.yexcr.2019.04.006 30978340

[82] Tu WJ McCuaig RD Tan AHY Hardy K Seddiki N Ali S. Targeting nuclear LSD1 to reprogram cancer cells and reinvigorate exhausted T cells via a novel LSD1-EOMES switch. Front Immunol. (2020) 11:1228. doi: 10.3389/fimmu.2020.01228 32612611 PMC7309504

[83] Chen Q Sun L Chen ZJ. Regulation and function of the cGAS-STING pathway of cytosolic DNA sensing. Nat Immunol. (2016) 17:1142–9. doi: 10.1038/ni.3558 27648547

[84] Wang M Li X Wu Y Wang L Zhang X Dai M. Loss of RPN1 promotes antitumor immunity via PD-L1 checkpoint blockade in triple-negative breast cancer - experimental studies. Int J Surg (London England). (2025) 111:1801–13. doi: 10.1097/js9.0000000000002164 39705151

[85] WangSZhangXChenQWuHCaoSZhaoS. FTO activates PD-L1 promotes immunosuppression in breast cancer via the m6A/YTHDF3/PDK1 axis under hypoxic conditions. J Adv Res. (2024), S2090-1232(24)00604-0. doi: 10.1016/j.jare.2024.12.026 39701379

[86] Xu H Guo Z Li M Chaves HV Pinto VDPT Filho GC. Copper-based nanomaterials for image-guided cancer therapy. Bio Integr. (2024) 5:1–14. doi: 10.15212/bioi-2024-0013

[87] Xu Y Zeng Y Xiao X Liu H Zhou B Luo B. Targeted imaging of tumor associated macrophages in breast cancer. Bio Integr. (2023) 4:114–24. doi: 10.15212/bioi-2022-0010

[88] Klinge CM. Non-coding RNAs in breast cancer: Intracellular and intercellular communication. Non-coding RNA. (2018) 4:40. doi: 10.3390/ncrna4040040 30545127 PMC6316884

[89] Li Z Yang HY Dai XY Zhang X Huang YZ Shi L. CircMETTL3, upregulated in a m6A-dependent manner, promotes breast cancer progression. Int J Biol Sci. (2021) 17:1178–90. doi: 10.7150/ijbs.57783 PMC8040468 33867838

[90] Wright J. Epigenetics: Reversible tags. Nature. (2013) 498:S10–1. doi: 10.1038/498S10a 23803942

[91] Garcia-Martinez L Zhang Y Nakata Y Chan HL Morey L. Epigenetic mechanisms in breast cancer therapy and resistance. Nat Commun. (2021) 12:1786. doi: 10.1038/s41467-021-22024-3 33741974 PMC7979820

[92] Schröder R Illert A-L Erbes T Flotho C Lübbert M Duque-Afonso J. The epigenetics of breast cancer – Opportunities for diagnostics, risk stratification and therapy. Epigenetics. (2022) 17:612–24. doi: 10.1080/15592294.2021.1940644 PMC9235902 34159881

[93] Neagu AN Whitham D Bruno P Morrissiey H Darie CA Darie CC. Omics-based investigations of breast cancer. Molecules. (2023) 28:4768. doi: 10.3390/molecules28124768 37375323 PMC10302907

[94] Kim K Skora AD Li Z Liu Q Tam AJ Blosser RL. Eradication of metastatic mouse cancers resistant to immune checkpoint blockade by suppression of myeloid-derived cells. Proc Natl Acad Sci U S A. (2014) 111:11774–9. doi: 10.1073/pnas.1410626111 PMC4136565 25071169

[95] Taylor K Loo Yau H Chakravarthy A Wang B Shen SY Ettayebi I. An open-label, phase II multicohort study of an oral hypomethylating agent CC-486 and durvalumab in advanced solid tumors. J Immunother Cancer. (2020) 8:e000883. doi: 10.1136/jitc-2020-000883 32753546 PMC7406114

[96] Yan Y Li S Su L Tang X Chen X Gu X. Mitochondrial inhibitors: a new horizon in breast cancer therapy. Front Pharmacol. (2024) 15:1421905. doi: 10.3389/fphar.2024.1421905 39027328 PMC11254633

[97] Terranova-Barberio M Thomas S Ali N Pawlowska N Park J Krings G. HDAC inhibition potentiates immunotherapy in triple negative breast cancer. Oncotarget. (2017) 8:114156–72. doi: 10.18632/oncotarget.23169 PMC5768393 29371976

[98] Christmas BJ Rafie CI Hopkins AC Scott BA Ma HS Cruz KA. Entinostat converts immune-Resistant breast and pancreatic cancers into checkpoint-Responsive tumors by reprogramming tumor-Infiltrating MDSCs. Cancer Immunol Res. (2018) 6:1561–77. doi: 10.1158/2326-6066.cir-18-0070 PMC6279584 30341213

[99] Ramaiah MJ Tangutur AD Manyam RR. Epigenetic modulation and understanding of HDAC inhibitors in cancer therapy. Life Sci. (2021) 277:119504. doi: 10.1016/j.lfs.2021.119504 33872660

[100] Wu HJ Chu PY. Epigenetic regulation of breast cancer stem cells contributing to carcinogenesis and therapeutic implications. Int J Mol Sci. (2021) 22:8113. doi: 10.3390/ijms22158113 34360879 PMC8348144

[101] Roussos Torres ET Rafie C Wang C Lim D Brufsky A LoRusso P. Phase I study of entinostat and nivolumab with or without ipilimumab in advanced solid tumors (ETCTN-9844). Clin Cancer Res. (2021) 27:5828–37. doi: 10.1158/1078-0432.ccr-20-5017 PMC8563383 34135021

[102] Jiang C Zhang S Jiang L Chen Z Chen H Huang J. Precision unveiled: Synergistic genomic landscapes in breast cancer-Integrating single-cell analysis and decoding drug toxicity for elite prognostication and tailored therapeutics. Environ Toxicol. (2024) 39:3448–72. doi: 10.1002/tox.24205 38450906

[103] Zhang H Lv G Liu S Liu D Wu X-Z. The artificial intelligence watcher predicts cancer risk by facial features. Tradit Med Res. (2022) 7:1. doi: 10.53388/tmr20211227255

[104] Karami Fath M Azargoonjahromi A Kiani A Jalalifar F Osati P Akbari Oryani M. The role of epigenetic modifications in drug resistance and treatment of breast cancer. Cell Mol Biol Lett. (2022) 27:52. doi: 10.1186/s11658-022-00344-6 35764927 PMC9238060

[105] Sher G Salman NA Khan AQ Prabhu KS Raza A Kulinski M. Epigenetic and breast cancer therapy: Promising diagnostic and therapeutic applications. Semin Cancer Biol. (2022) 83:152–65. doi: 10.1016/j.semcancer.2020.08.009 32858230

[106] Aafi E Reza M Mirabzadeh M. Jujube (Ziziphus jujuba Mill. (Rhamnaceae)): a review on its pharmacological properties and phytochemistry. Tradit Med Res. (2022) 7:31–40. doi: 10.53388/tmr20220905001

[107] Sarvari P Sarvari P Ramírez-Díaz I Mahjoubi F Rubio K. Advances of epigenetic biomarkers and epigenome editing for early diagnosis in breast cancer. Int J Mol Sci. (2022) 23:9521. doi: 10.3390/ijms23179521 36076918 PMC9455804

[108] Faldoni FLC Rainho CA Rogatto SR. Epigenetics in inflammatory breast cancer: Biological features and therapeutic perspectives. Cells. (2020) 9(5):1164. doi: 10.3390/cells9051164 32397183 PMC7291154

