# Correction: Epigenetic modifications in breast cancer: from immune escape mechanisms to therapeutic target discovery

**DOI:** 10.3389/fimmu.2025.1643911

**Published:** 2025-07-15

**Authors:** Ziyu Kang, Junlin Wang, Jiyan Liu, Li Du, Xiaofei Liu

**Affiliations:** ^1^ Institute of Chinese Medical Literature and Culture, Shandong University of Chinese Medicine, Jinan, China; ^2^ Department of Pharmacy, Shandong Second Provincial General Hospital, Jinan, China; ^3^ Pharmacy Department, Jinan Licheng District Mental Health Prevention and Control Center, Jinan, China; ^4^ Department of Acupuncture and Moxibustion, Shandong College of Traditional Chinese Medicine, Yantai, China; ^5^ Breast and Thyroid Surgery, Shandong University of Traditional Chinese Medicine Affiliated Hospital, Jinan, China

**Keywords:** breast cancer, epigenetic modifications, immune escape, multi-omics, therapeutic targets

In the published article, there was an error in affiliation 2. Instead of “Department of Pharmacy, The Second People’s Hospital of Shandong University, Jinan, China”, it should be “Department of Pharmacy, Shandong Second Provincial General Hospital, Jinan, China”.

The authors apologize for this error and state that this does not change the scientific conclusions of the article in any way. The original article has been updated.

